# Disability and health after replantation or revascularisation in the upper extremity in a population in southern Sweden – a retrospective long time follow up

**DOI:** 10.1186/1471-2474-15-73

**Published:** 2014-03-10

**Authors:** Hans-Eric Rosberg

**Affiliations:** 1 Department of Clinical Sciences Malmö – Hand Surgery, Skåne University Hospital, Lund University, 205 02 Malmö, Sweden

**Keywords:** Replantation, Hand trauma, Cold intolerance, Disability, Health outcome, Sense of coherence

## Abstract

**Background:**

Replantation in the upper extremity is a well-established microsurgical procedure. Many have reported patients’ satisfaction and functional measurements.

The aim was to investigate the long time consequences as activity limitations in hand/arm, the general health and cold sensitivity after a replantation or revascularization in the upper extremity and to examine if sense of coherence (SOC) can be an indicator for rehabilitation focus.

**Methods:**

Between 1994–2008, 326 patients needed replantation/revascularization in the upper extremity. 297 patients were followed up. Information was collected from the medical notes and by questionnaires [Quick-DASH (disability hand/arm), EuroQ-5D (general health), CISS (cold sensitivity) and SOC (sense of coherence)]. Severity of injury was classified with the modified Hand Injury Severity Score (MHISS).

**Results:**

The patients [272 (84%) men and 54 (16%) women; median age 39 years (1–81 years)], where most injuries affected fingers (63%) and thumb (25%), commonly affecting the proximal phalanx (43%). The injuries were commonly related to saws (22%), machines (20%) and wood splints (20%). A direct anastomosis (30%) or vein grafts (70%) were used. The overall survival was 90%. 59% were classified as Major.

Equal parts of the injuries took part during work and leisure, DASH scores at follow up were worse (p = 0.005) in the former. Twenty percent changed work and 10% retired early. Patients with early retirement were significantly older, had a more severe injury, worse disability, quality of life and functional outcome. Median DASH score was low [11.4 (0–88.6)] and correlated with severity of injury. Abnormal cold sensitivity (CISS > 50) was seen in 51/209 (24%) and they had a worse disability, quality of life, functional outcome and lower SOC. Patients with a low SOC had on the whole a worse outcome compared to patients with a high SOC and with significant differences in age, EQ-5D, Quick-DASH and CISS.

**Conclusions:**

A high MHISS, abnormal cold intolerance and a low SOC seems to be factors influencing the patients’ outcome and might be relevant in the rehabilitation of the patients. Also, those who had to retire early had a worse disability, quality of life and functional outcome.

## Background

Replantation and revascularization in the upper extremity after severe hand injury or total or subtotal amputation of fingers or, hand or arm is a well established microsurgical procedure [1]. Many reports describe the outcome of digital replantation and reporting survival rates and functional outcome [2-10], including thumb replantation [11-13] and more proximal replantations, have been validated [14,15]. Most of the studies report overall survival rates and have used some classification system to report functional outcome. There is a discrepancy between the subjective satisfaction from the surgery and objective measurements of range of finger motion and grip strength [8-10,15]. The patient’s real benefit from a replantation is difficult to define. It may depend on how well the patient can cope with the trauma and be able to return to normal life of daily living, work and leisure activities.

The SOC instrument reflects a person’s capacity to respond to a stressful situation [16,17]. In patients with moderate orthopaedic injuries, like different types of fractures in the lower and upper limb, it has been shown that low SOC is associated with an increased risk of having less good clinical and functional outcome [18].

The purpose of this study is to evaluate the long time outcome in hand function, health and quality of life, cold sensitivity and sense of coherence.

## Methods

Between January 1994 and December 2008, 326 patients had surgery due to no arterial circulation in a finger, hand or arm. At follow up fourteen had died, 11 were foreigners (i.e. no contact address) and 4 had no address. Left for follow up were 297 patients. Ethical approval was obtained from the ethics committee Malmö/Lund, Sweden (ethical permit number 714/2004).

Information regarding the accident and surgery was found in the medical notes. Age at injury, sex and work was also found in the medical notes.

The severity of the injury was classified with the modified Hand Injury Severity Score (MHISS) [19]. The injuries can then, depending on the score, be divided into broad categories, such as “Minor”, “Moderate”, “Severe” and “Major”. With the HISS it is only possible to score injuries distal to the wrist. The MHISS will make it possible to include injuries proximal to the carpus and in the forearm. However, still is it not possible to score injuries in the upper arm. In this study, only three patients had an injury in the upper arm and therefore an approximated score for those patients with an injury in the upper arm was calculated.

Questionnaires were sent to the patients between 3 and 17 years (median 10 years) after the injury. Two hundred fourteen patients answered the questionnaires (72%). Seventy two per cent of the patients responded; response was not e.g. by the youngest and oldest patients. The questionnaires were: the Disability of the Arm, Shoulder and Hand (DASH), EuroQol (EQ-5D), Cold Intolerance Symptom Severity (CISS), a questionnaire about diseases and work for the patient and Sense Of Coherence (SOC) [16,20-22].

### DASH

DASH is a region specific instrument [23,24]. The questionnaire covers daily activities, symptom questions and questions related to self-image and social functioning. The DASH score ranges from 0 to100; 0 indicating no disability and 100 signifying the most severe disability. In this study the Quick-DASH version was used [20].

### EQ-5D

To measure health outcome in different groups, the EQ-5D was used [21,25]. It is composed of five dimensions: mobility, self-care, usual activities, pain/discomfort and anxiety/depression. The reduction in quality of life associated with the health state was taken from the Danish TTO-tariffs [26] and defined as the difference between perfect health and the health state value corresponding to the EQ-5D code. The summary index score of 1 represent the best possible state of health and 0 represent the worst condition. EQ-5D also has a visual analogue scale (EQ-5D VAS). The patient rates his or her current health statues on a VAS scale; a higher score indicates a better health (0–100). The VAS scale generates more general information on the self-perceived health state than the EQ-5DIndex with its five dimensions.

EQ-5D is an international, widely used questionnaire for measuring health related quality of life for different patient categories. EQ-5D has been used to score quality of life for hand injuries, but not for replanted patients [27-29]. It is validated and it is reliability has been tested [30]. It is easy to complete and quick to interpret.

### SOC

Sense of coherence (SOC) was assessed with Antonovsky’s short 13-item scale. The scores on each item ranges from 1 to 7 with a total score range from 13–91. High scores indicate a high SOC. The SOC instrument reflects a person’s capacity to respond to a stressful situation and according to Antanovsky a person’s individual environment as being a comprehensible, manageable and meaningful [16,17]. According to Eriksson and Lindström, SOC seems to be a health promoting resource [31] influencing quality of life [32]. Higher SOC has also been reported to result in a more positive development of life satisfaction [33]. In patients with moderate orthopaedic injuries, it has been shown that low SOC was associated with an increased risk of having less good clinical and functional outcome [18]. Normative data from published studies ranges from mean values 58.5 to 68.7.

### CISS

The Swedish version of the CISS questionnaire was used [22]. Question number 1 concerns severity of different symptoms on exposure to cold and is not included in the total CISS score and has a numeric rating scale (0 = no symptoms/trouble at all and 10 = the most severe symptoms/trouble you can possible imagine).

### Functional evaluation

A part of the patients (n = 194) had been for an examination to issue a certificate for an insurance company. Information was collected from the certificate, regarding grip power, pinch grip power, range of motion and daily activity. The examination for the certificate was done between one and 10 years after the injury (median 2 years).

### Subgroups

The whole group were divided into subgroups with respect to severity of injury (MHISS), cold sensitivity (CISS), return to work and sense of coherence (SOC). The injury severity was classified with MHISS and depending on the score the population was divided into three groups “Moderate”, “Severe” and “Major”. Abnormal cold sensitivity was defined as the cut-off value by Carlsson, CISS score >50 = abnormality and was used to dichotomize the study population [34]. The working group of people was dichotomized into one group who went back to work and one who had to retire early. The median SOC score of 75 was used to dichotomize the study population into low SOC and high SOC.

### Data analysis

All variables are presented as median (min-max). Kruskal Wallis test or Mann–Whitney *U*-test was used to detect differences between subgroups. Fisher’s exact test was used in analysis of contingency tables. The significance level was set at p ≤ 0.05.

## Results

### General information

Table 1 summarises some variables for the total number of patients and also divided into groups with different replantation/revascularization level. Equal parts took place during work (50%) and leisure (50%). More men were injured at work (54%) than women (31%). Avulsion injuries and log splitter injuries were more common among women (19% and 30%) than men (9% and 18%). The most common injuries in men were by a saw (26%) and a crush injury (14%) compared to women (4% and 8%). The injury level was the same in men and women with fingers and thumb as most common.

**Table 1:** Total number of patients divided into groups with different levels of replantation/revascularization

	**Total n = 326**	**Thumb n = 83**	**Finger n = 205**	**Middle hand n = 14**	**Wrist n = 9**	**Proximal to wrist n = 13**
Age (years)	39 (1–81)	46 (8–81)	34 (1–76)	30,5 (9–63)	30 (18–66)	49 (8–68)
Gender F/M	17%/83%	16%/84%	17%/83%	14%/86%	22%/78%	15%/85%
Cause of injury(%)						
Saw	**22**	**29**	**22**	**21**		
Wood cutter	**20**	**18**	**21**	**21**	**11**	**31**
Machine	**20**	**23**	**18**	**21**	**22**	
Farmer					**33**	**15**
Crush						**23**
HISS	120 (24–409)	144 (78–406)	84 (24–409)	174 (56–337)	200 (107–323)	190 (100–250)
EQ-5D VAS	80 (5–100)	80 (30–100)	80 (12–100)	80 (15–90)	70 (5–78)	80 (10–100)
EQ-5D	0.824	0.824	0.824	0.824	0.708	0.824
index	(-0.624 -1.0)	(-0.324 –1.0)	(-0.624 –1.0)	(0.075 – 1.0)	(0.034–0.713)	(0.374 – 1.0)
QuickDASH	11.4 (0–88.6)	9.1 (0–72.7)	9.1 (0–88.6)	13.6 (2.3-47.7)	43.2 (27.3-88.6)	29.5 (0–59.1)
CISS	36 (10–89)	35 (4–75)	35 (0–89)	37 (14–82)	44 (18–52)	41 (4–56)
SOC	75 (27-93	76 (50–90)	73 (27–93)	79 (40–91)	72 (68–84)	80 (28–91)
ADL	13 (10–25)	13 (10–21)	13 (10–24)	14 (10–22)	21 (14–25)	18 (14–21)
JAMAR% of contalateral	66 (0–100)	84 (24–100)	64 (2–100)	56 (7–100)	28 (0–51)	25,5 (0–75)
PINCH% of contralateral	75 (0–100)	56 (12–100)	80 (6–100)	62 (9–100)	31 (12–62)	40,5 (0–80)

Median and min – max values.

Bold indicate significant difference.

**Figure 1:**
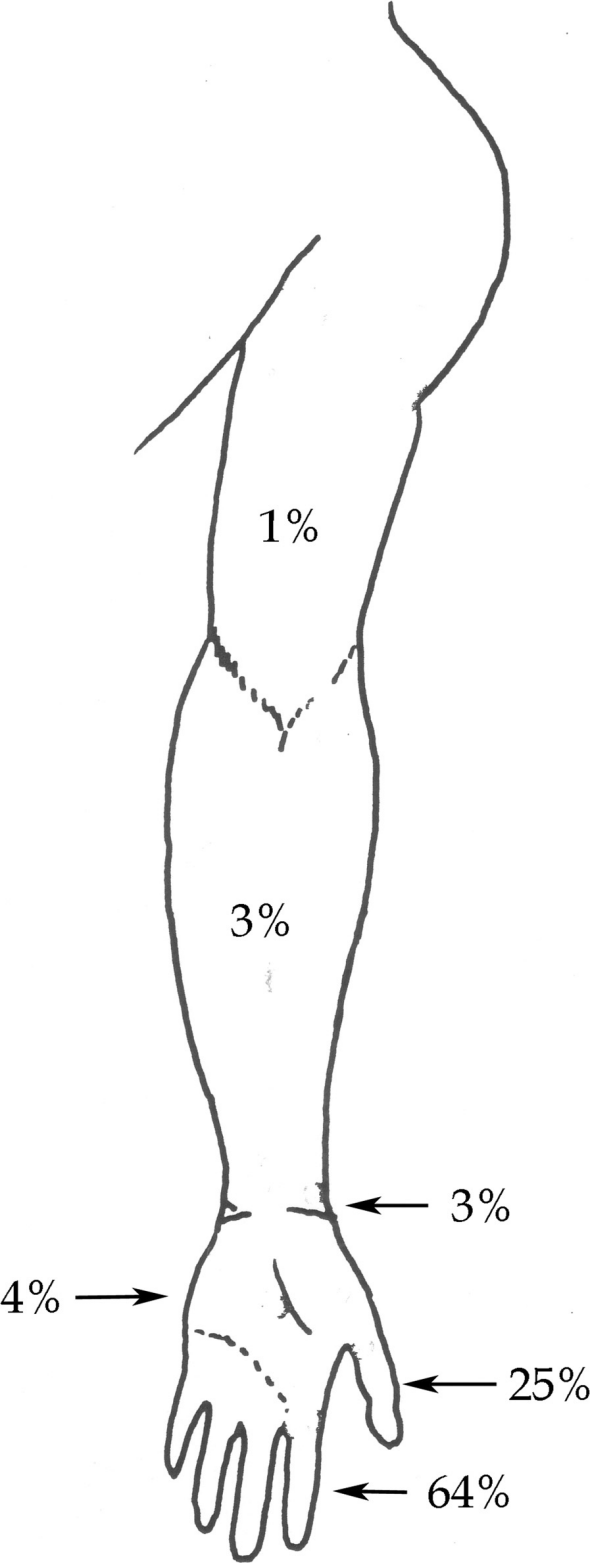
Level of amputation in the upper extremity and the level of amputation in the hand expressed in per cent.

The level of injuries is presented in Figure 1. The total number of injured fingers/thumbs was 680 (242 fingers not needing replantation/revascularization) and the numbers of replanted fingers and thumbs were 350 and 88, respectively. When comparing finger and thumb replantation, we found that the patients with a thumb replanted were older (p = 0.0002) and had a higher HISS (p = <0.0001) than the finger replanted. The grip strength (p = 0.001) was lower in the group of finger replanted and the pinch strength (p = 0.004) was lower in the group with thumb replantation. The most frequent replanted finger was index (n = 110) and long finger (n = 103).

Sixty per cent were a replantation and 40% were a revascularisation. The overall survival rate was 90%. The survival rate for fingers was 91% and for thumbs 86%. In 70% of the patients a vein graft was used and in 30% a direct anastomosis was sufficient.

The replanted patients had a higher HISS (p < 0.0001) than those who had a revascularization.

### Severity of hand injury

The median HISS was 120 (24–406) and were classified as Moderate (13%), Severe (28%) and Major (59%). When comparing the severity groups those with a Major injury were older than those with a Severe injury (Table 2). Those with a Major injury also had a worse QuickDASH, CISS, ADL, grip strength and pinch strength compared with both Severe and Moderate injuries (Table 2). A significant difference was also found between Major and Moderate injuries for EQ-5D Index and SOC and also between Severe and Moderate injuries for SOC. No difference in EQ-5D VAS was found (Table 2).

**Table 2:** Differences between patients with different hand injury severity according to HISS severity groups

	**Moderate**	**Severe**	**Major**	**P-values**
	**(n = 41)**	**(n = 92)**	**(n = 192)**	
**Age**	35	34▲	41	0,002
EQ-5D VAS	84	79	74	0,09
**EQ-5D Index**	0,874†	0,809	0,760	0,02
**DASH**	8,9†	15,5▲	21,5	0,004
**CISS**	31†	32▲	41	0,002
**SOC**	79†ϒ	70	71	0,04
ADL*	12,5†	12,6▲	14,6	<0,0001
JAMAR%*	79†	68▲	57	0,0007
PINCH%*	79†	76▲	61	0,001

*Moderate (n = 24), Severe (n = 54) and Major (n = 116).

†Significant different from Major.

ϒSignificant different from Severe.

▲Significant different from Major.

### Cold sensitivity

The median CISS was 35 (0–89). Abnormal cold sensitivity (CISS > 50) was seen in 24% (51/209). The predominant problems on exposure to cold among the patients were numbness, stiffness, weakness, skin colour change and pain and aching.

Those with an abnormal cold sensitivity had a worse QuickDASH, ADL, grip strength and pinch strength. A significant difference was also found for EQ-5DIndex and EQ-5DVAS. They also had a lower SOC (Table 3).

**Table 3:** Differences between patients with an abnormal cold intolerance (CISS > 50) and those with CISS ≤50

	**CISS ≤50 (n = 161)**	**CISS > 50 (n = 51)**	**P-values**
Age	38	43	0.07
HISS	134	160	0.06
**EQ-5D VAS**	80	66	<0.0001
**EQ-5D Index**	0.831	0.690	<0.0001
**Quick-DASH**	13	33	<0.0001
ADL*	14	15	0.03
**SOC**	74	64	<0.0001
JAMAR%*	67	52	0.01
PINCH%*	74	47	<0.0001

*CISS ≤ 50 (n = 102) and CISS >50 (n = 34).

### Early retirement

Information regarding the patients work situation at time of injury was available in 268 patients. Most of the patients had a work at the time of injury (n = 206), 40 were school children or students, 6 were preschool children and 16 were retired. Twenty-two per cent of the patients who worked at time of injury had to change work and 10% had to retire early due to the injury.

If the injury took place at work the DASH was worse (p = 0.005) than if the injury were sustained at leisure. Also the grip strength (p = 0.03) and SOC (p = 0.04) was lower in the group injured at work.

Twenty-one patients were not able to return to work. This group was significantly older and had a higher HISS. They had a worse disability (DASH), worse daily occupation (ADL), worse cold sensitivity (CISS) and also a worse health outcome (EQ-5D VAS and EQ-5D Index. They had a worse grip and pinch strength (Table 4).

**Table 4:** Differences between patients who went back to work and those who had to retire early due to the injury

	**Back to work (n = 185)**	**Early retirement (n = 21)**	**P-values**
**Age**	37	52	<0,0001
**HISS**	133	194	0,002
**EQ-5D VAS**	79	62	0,01
**EQ-5D Index**	0,821	0,629	0,008
**Quick-DASH**	13,9	45,2	<0,0001
**CISS**	35	57	<0,0001
ADL*	14	16	0,003
SOC	72	67	0,08
JAMAR%*	67	31	<0,0001
PINCH%*	69	46	0.01

*Back to work (n = 115) and Early retirement (n = 15).

### Sense of coherence

Between the subgroups with high or low SOC, significant differences in outcome were found. Patients with a low SOC had on the whole a worse outcome compared to patients with a high SOC. A significant difference was found in age between the groups, but no difference in the severity of hand injury (Table 5). EQ-5D VAS, EQ-5D Index, Quick-DASH, CISS and ADL-questionnaire showed better values in patients with high SOC (Table 5). No significant difference was found in grip strength and pinch strength (Table 5).

**Table 5:** Differences between patients with high (≥75) or low (<75) SOC

	**High SOC (n = 107)**	**Low SOC (n = 104)**	**P-values**
**Age**	42	36	0.004
HISS	139	140	0.9
**EQ-5D VAS**	84	68	<0.0001
**EQ-5D Index**	0.865	0.712	<0.0001
**Quick-DASH**	12.7	24.1	<0.0001
**CISS**	31	43	<0.0001
ADL*	13.4	14.7	0.01
JAMAR%*	64	62	0.6
PINCH%*	68	66	0.5

*High SOC (n = 58) and Low SOC (n = 75).

## Discussion

Complete or subtotal amputation injuries requiring revascularisation or replantation have a severe impact on the individual patient and may profoundly influence his or her life due to limitations of function. Despite this and interestingly, most of the present patients were able to return to some work activity. They also valued their symptoms and ability to perform daily activities as manageable. Only 10% did not return to work, although 22% had to change work. Interestingly, those with a low SOC had worse health outcome, worse disability, more severe cold intolerance and more problems with daily activities, which is in accordance with a a study of major hand injuries [35]. This might indicate a group of patients that may need more attention during rehabilitation.

### General information

The most frequent causes of injury were by saw, woodcutter or a machine, except if the injury was at the wrist level or more proximal. In these cases, it was still from a woodcutter, but also from farmer, machine or crush (Table 1). In this study, 50% of the injuries were work-related, which is lower than have been reported earlier [36].

The overall success rate was 90%, which has been reported by others [8,12,37], indicating that the present material is valid. Survival rates for digital replantation have been analyzed in a meta-analysis and a clear difference exists between a clean-cut injury (91%) and crush (68%) or avulsion (66%) injuries [37]. Also, the level of amputation has been found to influence the survival rate [12,14,38]. The high success rate might depend on our preoperative selection of patients suitable for replantation.

### Injury severity

Patients needing replantation is a very heterogeneous group from an almost surgical clean cut amputation of a thumb to a patient with multiple injured fingers, but only the thumb needs revascularisation. To be able to reflect this situation, the MHISS was used to score the total severity of the injury. Not only the finger(s) needing revascularization, but the complexity of the total injury influence the outcome and how the patients deal with daily living, work and leisure. MHISS correlated with QuickDASH, which is in accordance with earlier studies [39,40]. It may indicate that a more severe injury needs more rehabilitation resources to gain a more useful hand function. Tamai’s score system, which is often used in replantation studies, has also been found to correlate with HISS [40].

### Cold intolerance

Cold intolerance is a common problem after replantation and known to be a longstanding problem [28]. However, different classification systems have been used and therefore different degrees of cold intolerance are reported [2,9,14]. We used the Swedish CISS questionnaire, which is reliable and validated [22].

All patients in the present study reported some type of cold intolerance. Abnormal cold sensitivity influences the patient’s daily life, work and leisure. When using the cut of value by Carlson et al. [34] for defining an abnormal cold sensitivity 24% had an abnormal cold sensitivity which is in accordance with others [41]. A high correlation between CISS and hand disability, quality of life and functional outcome was also found. They also had a low SOC. This is information is important to the rehabilitation team in helping the patient with protection and rehabilitation strategies for handling this difficult problem.

### Disability

The DASH and also Quick-DASH was designed to assess upper extremity disability and symptoms and could be helpful in evaluating the effect of a treatment on the upper limb and serves as a value with which other outcomes can be composed. DASH or Quick-DASH have been used in other replantation studies and reported values are the same as in this study [2,13]. However, other reported higher DASH scores, which might reflect different follow up times and different patient selection [11,42].

### Quality of life

Only in one previous study [15] quality of life has been investigated in replanted patients. However, the EQ-5D has not before been used in this patient group. Hand function has been evaluated previously by different types of scores [2,8,11,13,14,42]. Quality of life is more than hand function. Both EQ-5DIndex and EQ-5D VAS correlated with QuickDASH, but not with the severity of injury. The EQ-5D Index and EQ-5D VAS scales have been shown to be age dependent [43,44], which was not confirmed.

### Return to work

One third of the injured patients, who had a work, had to change work or retire early due to the injury. They were older and had a more severe injury, indicated by a higher HISS, and were more often injured at work. They also had more problems with daily activities and a worse quality of life. Also, functional variables, as grip- and pinch strength, were worse. No difference was found between men and women. Almost comparable figures for return to work have been shown by others [11,13,14].

Return to work in a group of patients with a serious hand injury was not related to the severity of the hand injury (HISS) [45]. However, it seemed that factors more dependent on the person’s own ability and motivation was important. This may indicate that SOC could play an important role for how a patient will be able to rehabilitate after a hand injury.

In contradiction, other has reported that HISS could adequately be used to predict return to work [40]. In this study, also age and the place of injury might have influenced return to work. An impaired hand function, such as cold sensitivity [46], pain [47] and reduced grip strength [48,49], can greatly hinder recovery and reduce successful functional outcome and return to work, which was confirmed. The ability to return to work is probably not only dependent on the above factors, but also on the socioeconomic benefits of the given society.

### Sense of coherence

Several differences in outcome between patients with a high or low SOC were found. The relationship between the SOC and outcomes after amputation injuries in the upper extremity, needing replantation or revascularisation, has not been previously studied. In a recently published study, patients with a severe or major hand injury and a low SOC showed significantly lower satisfaction in daily occupation, higher DASH scores, lower mental QoL, more sleep disturbances and bodily pain [35]. Also, after moderate orthopaedic injuries, a low SOC were associated with an increased risk of having a less good clinical and functional outcome [18]. There are some problems with SOC, such as the influence of age [17], different life situations effect [33,50] and the interpretation of healthy/unhealthy [35]. Another possible limitation with SOC might be that no preinjury value is available. As a cut point the median value of SOC could not be considered a generalized recommended cut point for screening individuals. The findings, like others [35], suggest that SOC need to be considered further when planning rehabilitation of patients with severe hand injuries.

## Conclusions

On the whole, the majority of replanted patients did well. Only 10% were not able to return to any work. Cold intolerance is still a major problem, which may give long-standing problems. The disability correlated with the injury severity and a more severe injury had a worse quality of life and functional outcome. Patients with low SOC had a worse outcome compared with a high SOC, indicating that this group of patients may benefit from more meticulous and guided rehabilitation. In the future, we need to have information regarding the patients coping ability when designing rehabilitation programs for replantation patients.

## Abbreviations

CISS: Cold intolerance symptom severity; EuroQ-5D: EuroQol-5 Dimension; MHISS: Modified hand injury severity score; QuickDASH: Quick disability of the arm, shoulder and hand; SOC: Sence of coherence.

## Competing interests

The author declares that he has no competing interests.

## Authors’ contributions

The author has design the study, coordinated the study, performed the statistical analyses and drafted the manuscript.

## Pre-publication history

The pre-publication history for this paper can be accessed here:

http://www.biomedcentral.com/1471-2474/15/73/prepub
